# Strategies to address reproductive coercion and intimate partner violence in Nairobi family planning services: qualitative client and provider perspectives

**DOI:** 10.1080/26410397.2025.2570528

**Published:** 2025-10-10

**Authors:** Jasmine Uysal, Emilie Schwarz, Wilson Liambila, Seri Wendoh, Ruvani W. Fonseka, Ricardo Vera Monroy, Sabrina C. Boyce, Erin Pearson, Jay G. Silverman

**Affiliations:** a Doctoral Candidate, Center on Gender Equity and Health, University of California San Diego, La Jolla, CA, USA.; b Student Researcher, Center on Gender Equity and Health, University of California San Diego, La Jolla, CA, USA; c Program Associate, Population Council – Kenya, Nairobi, Kenya; d Global Lead Gender & Inclusion, International Planned Parenthood Federation, London, UK; e Instructor, Department of Community and Public Health, City College of San Francisco, CA, USA; f Data Analyst & Systems Manager, School of Public Health, University of California, Berkeley, CA, USA; g Assistant Professor, School of Public Health, University of California, Berkeley, CA, USA. boyce.sabrina@berkeley.edu; h Director of Global Partnerships and Collaborative Learning, Center on Gender Equity and Health, University of California San Diego, La Jolla, CA, USA; i Professor, Weatherhead Tulane School of Public Health and Tropical Medicine, New Orleans, LA, USA

**Keywords:** reproductive coercion, reproductive autonomy, sexual and reproductive health and rights, intimate partner violence, gender-based violence, violence prevention, implementation, qualitative, family planning

## Abstract

Reproductive coercion (RC) and intimate partner violence (IPV) threaten women’s health and reproductive agency globally. The Addressing Reproductive Coercion in Health Settings (ARCHES) intervention integrates screening for RC and IPV, covert contraceptive options, IPV referrals, and an educational booklet into family planning (FP) services. This study examines client and provider perspectives on ARCHES in Nairobi, Kenya. A qualitative study was conducted in the three private clinics implementing ARCHES. Data were collected through 35 in-depth client interviews and 12 provider interviews three months post-intervention. Participants were purposively sampled based on age and RC/IPV experience. Thematic content analysis was used. Clients reported greater reproductive autonomy through increased knowledge of covert contraception, IPV services, and reproductive rights. Providers reported gaining confidence to address RC and IPV and building stronger relationships with clients. Barriers included stigma around IPV referrals and limited counselling time. Screening for RC/IPV was widely accepted, with some clients recognising abuse for the first time. The compact educational booklet improved knowledge retention and community outreach. Perspectives on male partner engagement were mixed due to concerns about safety and confidentiality. ARCHES was well-received by clients and providers, enhancing FP care and addressing critical gaps in RC and IPV support. Future adaptations should address barriers to IPV referral uptake and provider first-line IPV support, and carefully track implementation to ensure continued client-centred counselling. These findings support ARCHES as a scalable model for integrating FP and violence prevention interventions in Kenya and similar contexts.

## Trial registration

ClinicalTrials.gov identifier: NCT03534401.

## Plain language summary

Reproductive coercion (RC) and intimate partner violence (IPV) threaten women's health worldwide. The ARCHES programme was designed to support women in family planning (FP) clinics by addressing RC and IPV, offering discreet contraception options, providing IPV referral, and distributing an educational booklet. Healthcare providers were also trained to prioritise client privacy and offer women the choice to involve their male partners in FP discussions. This study examined how ARCHES was received by clients and providers in private clinics in and around Nairobi, Kenya. We conducted interviews with 35 clients and 12 providers three months after implementation. Clients said they felt empowered by learning about discreet contraception options, IPV services, and their reproductive rights. This helped them make more informed FP decisions. Providers gained confidence in addressing RC and IPV, strengthening their relationships with clients. Continuing challenges included barriers to IPV referrals and limited time for counselling. Screening for RC and IPV was widely accepted, with many clients recognising abuse for the first time and feeling supported. The compact educational booklet helped women remember what they had learned from the provider and share information within their communities. Some clients and providers expressed concerns about safety and confidentiality if male partners were involved. However, the overall reception of ARCHES was positive. The programme improved FP care by addressing critical gaps in RC and IPV support. Future adaptations should focus on (1) making IPV referral more acceptable, (2) strengthening provider training, and (3) ensuring counselling is client-centred. These findings suggest ARCHES is a scalable model for integrating FP and gender-based violence interventions in Kenya and similar settings.

## Résumé

La coercition reproductive et la violence exercée par un partenaire intime (VPI) menacent la santé et l’autonomie reproductive des femmes dans le monde. L’intervention ARCHES (Addressing Reproductive Coercion in Health Settings/S’attaquer à la coercition reproductive dans le domaine de la santé) intègre le dépistage de la coercition reproductive et de la VPI, des options contraceptives dissimulées, l’orientation vers les services compétents en cas de VPI et une brochure pédagogique sur les services de planification familiale. Cette étude examine les points de vue des clientes et des prestataires sur ARCHES à Nairobi, au Kenya. Une étude qualitative a été menée dans les trois centres privés mettant en œuvre l’initiative ARCHES. Les données ont été recueillies au cours de 35 entretiens approfondis avec des clientes et de 12 entretiens avec des prestataires trois mois après l’intervention. L’échantillon des participantes a été sélectionné à dessein en fonction de leur âge et de leur expérience en matière de coercition reproductive et de VPI. Une analyse thématique du contenu a été réalisée. Les clientes ont déclaré avoir accru leur autonomie reproductive grâce à une meilleure connaissance de la contraception dissimulée, des services de VPI et des droits reproductifs. Les prestataires ont indiqué qu’ils avaient gagné en confiance pour aborder la coercition reproductive et la VPI ainsi que pour nouer des relations plus fortes avec leurs clients. Les obstacles comprenaient la stigmatisation autour de l’orientation en cas de VPI et le temps limité consacré aux conseils. Le dépistage de la coercition reproductive et la VPI était largement accepté, certains clientes reconnaissant des violences pour la première fois. La brochure pédagogique a amélioré la rétention des connaissances et la sensibilisation communautaire. Les points de vue sur l’engagement des partenaires masculins étaient mitigés en raison de préoccupations liées à la sécurité et la confidentialité. L’initiative ARCHES a été bien reçue par les clientes et les prestataires, améliorant les soins de planification familiale et comblant les lacunes importantes dans le soutien en cas de coercition reproductive et de VPI. Les futures adaptations devraient lever les obstacles à l’orientation vers les services de VPI et au soutien de première ligne des prestataires en cas de VPI, et suivre attentivement la mise en œuvre afin de garantir la poursuite d’un accompagnement centré sur les clientes. Ces résultats confirment qu’ARCHES constitue un modèle évolutif d’intégration des interventions de planification familiale et de prévention de la violence au Kenya et dans des contextes similaires.

## Resumen

La coerción reproductiva (CR) y la violencia de pareja íntima (VPI) amenazan la salud y la agencia reproductiva de las mujeres a nivel mundial. La intervención Abordaje de la Coerción Reproductiva en Ámbitos de Salud (ARCHES, por sus siglas en inglés) integra en los servicios de planificación familiar (PF) la detección de CR y VPI, opciones anticonceptivas discretas, referencias de casos de VPI y un folleto educativo. Este estudio examina las perspectivas de usuarias y prestadores de servicios sobre ARCHES en Nairobi, Kenia. Se realizó un estudio cualitativo en las tres clínicas privadas que estaban implementando ARCHES. Se recolectaron datos por medio de 35 entrevistas a profundidad con usuarias y 12 entrevistas con prestadores de servicios tres meses después de la intervención. Los participantes fueron muestreados a propósito según su edad y experiencia de CR/VPI. Se utilizó el análisis de contenido temático. Las usuarias relataron una mayor autonomía reproductiva mediante mayor conocimiento del uso encubierto de anticonceptivos, servicios de VPI y derechos reproductivos. Los prestadores de servicios relataron adquirir confianza para abordar la CR y la VPI y forjar relaciones más sólidas con las usuarias. Entre las barreras figuraban el estigma en torno a las referencias de VPI y tiempo limitado para la consejería. La detección de CR/VPI fue aceptada de manera generalizada, y algunas usuarias reconocieron su maltrato por primera vez. El folleto educativo conciso mejoró la retención de conocimiento y la extensión comunitaria. Las perspectivas sobre la participación de parejas masculinas eran mixtas debido a las preocupaciones sobre la seguridad y confidencialidad. ARCHES fue bien recibida por las usuarias y los prestadores de servicios, y mejoró los servicios de PF y abordó las brechas críticas en el apoyo a quienes sufren CR y VPI. Futuras adaptaciones deben abordar las barreras a la aceptación de referencias de VPI y el apoyo de prestadores de servicios de primera línea a víctimas de VPI, y deben llevar un registro cuidadoso de la implementación para garantizar una continua consejería centrada en las usuarias. Estos hallazgos respaldan ARCHES como un modelo ampliable para integrar intervenciones de PF y de prevención de violencia en Kenia y en contextos similares.

## Introduction

Reproductive coercion (RC) and intimate partner violence (IPV) are forms of gender-based violence (GBV) that contribute to unmet need for family planning (FP) and unintended pregnancy worldwide.^1^ RC describes abusive behaviours that undermine women’s reproductive agency by controlling contraceptive or pregnancy decisions. These behaviours may promote or prevent pregnancy and include threats or pressure to become pregnant (pregnancy coercion), restricting or forcing abortions (abortion coercion), interference with contraceptive use (contraceptive sabotage), and coerced contraceptive use (forced use of contraception or sterilisation).^1–4^ One of the primary ways women maintain reproductive agency in the face of RC is to use contraceptives without the knowledge of their male partners and/or family members, otherwise known as covert contraceptive use.^5–7^

IPV describes abuse by a former or intimate partner, including physical, sexual, or emotional harm.^8^ RC and IPV often co-occur and are both linked to negative health outcomes, such as unintended pregnancy, induced abortion, and rapid repeat pregnancies.^1,2,4,9,10^ Globally, one in four women will experience IPV; reports of RC range from 3% to 42%, depending on the context.^11,12^ Women seeking reproductive health services report higher rates of gender-based violence (GBV) than the general population, emphasising the need to address violence in this setting.^13,14^ In 2019, the World Health Organization included identification and support for both IPV and RC survivors in healthcare guidelines.^8,15^

Like other contexts, RC and IPV contribute to poor reproductive health outcomes in Kenya. Two in five women report lifetime IPV, and one in three in the past year.^16^ According to the 2022 Kenya Demographic and Health Survey (DHS), 10.9% of women currently partnered reported lifetime pregnancy coercion. One in three births is unintended.^16^ The Kenyan Ministry of Health has prioritised reducing unmet need for contraceptives and addressing unintended pregnancies and violence.^17^ No clinic-based interventions, however, have existed in Kenya to address RC and IPV within contraceptive services prior to the intervention described here.

Addressing Reproductive Coercion in Health Settings (ARCHES) is an evidence-based intervention that trains existing family planning providers on strategies to address IPV and RC within routine family planning counselling. In this paper, we describe client and provider perspectives of ARCHES as delivered from three private facilities in and around Nairobi.

## The ARCHES approach

The ARCHES approach was initially developed and tested in the United States (US) through two cluster randomised controlled trials, which showed reduced experiences of RC and improved coping with IPV.^14,18–20^ ARCHES aims to increase women’s agency to control their FP and pregnancy decisions by addressing RC and IPV through three key strategies, integrated into standard family planning counselling, including: (1) providing information and screening on RC, including contraceptive methods that can be used covertly, (2) screening for IPV with a supportive response and, for those disclosing IPV, offering a supported referral to local IPV support services; and (3) distributing palm-sized educational booklets on RC, IPV, contraceptive methods, and referral contacts. Core values of the intervention emphasise women’s right to confidential reproductive health care and control over their contraceptive and pregnancy decisions, including covert contraceptive use, if desired.

In 2015, the model was adapted for private family planning clinics in Nairobi, Kenya, run by Family Health Options of Kenya (FHOK), using formative research with clients and providers in a participatory process involving clinic leadership and providers.^21^ To further protect women’s confidentiality in this context where male engagement was highly encouraged,^22^ the Kenyan adaptation included protocols to protect female clients' privacy from accompanying male partners or family members. Women were also offered the option to invite their partners back to the clinic for additional information if they found it helpful, without pressure to do so.^21^

After a three-month pilot, ARCHES was evaluated in a non-randomised, cluster-controlled trial with 659 women and girls seeking family planning in six private clinics (three ARCHES, three standard-of-care) in and around Nairobi. Client surveys taken immediately after receiving services showed high rates of fidelity. For each strategy, participants indicated a reception rate ranging from 85% to 95%; 77% received all four components. Among women reporting RC or IPV at baseline, over 7 out of 10 disclosed to their provider, and nearly all (98%) women accepted the palm-sized, educational booklet. The evaluation found ARCHES, as compared to standard family planning counselling, increased contraceptive uptake, awareness of IPV services, and decreased attitudes supportive of RC, incident pregnancy, and physical intimate partner violence.^23,24^

## Methods

### Study design

To contextualise quantitative results and implementation of ARCHES, 35 intervention participants reporting prior RC, stratified by age (15–24 and 25–49) and prior experience of IPV, were purposively selected and invited by phone for in-depth interviews three months after their initial appointment. Interviews focused on clients’ perceptions of and experiences with the integrated ARCHES approach, including changes in their lives due to the programme. Additionally, 12 providers trained in ARCHES who confirmed providing ARCHES to at least 25 clients over the implementation period were recruited by the local project coordinator to participate in post-implementation interviews approximately three months post-training. No clients nor providers refused to participate. The sample size was chosen to achieve thematic saturation while considering available resources.

Interview tools were collaboratively developed by team members in the US and Kenya based on research questions from the parent study. Guides were pretested during interviewer training, and refinements were made. The final semi-structured interview guide for providers included 13 open-ended questions on the feasibility of delivering ARCHES, its acceptability to providers and clients, attitudes towards women using contraception covertly, and suggestions for improving implementation, including post-training support. The client guide included 15 open-ended questions about their experiences with ARCHES, its influence on their contraceptive use or relationships, whether they shared the information they received, and recommendations for improving FP care.

Data were collected between December 2018 to March 2019 by two female research assistants with master’s-level training and mentorship in qualitative research from the site principal investigator (PI). Participants were introduced to interviewers by members of the research team who collected their baseline and exit surveys from the parent study. Interviews were conducted in Kiswahili at family planning clinics, in private locations, and lasted 60–90 minutes. Interviews were recorded, transcribed into English, and anonymised with participant numbers containing only age group and gender. Field notes were taken by the interviewer after the interview. No repeat interviews were conducted, and transcripts were not returned for participant review. No one else besides the interviewer and participant was present during data collection.

### Ethics

This study was approved by the University of California San Diego’s Human Research Protections Program (Protocol 170084, February 7, 2017), the Population Council Institutional Review Board (Protocol 797, January 18, 2017), and the Kenyatta National Hospital-University of Nairobi Ethics and Research Committee (Protocol P945/12/2016, March 2, 2017). All participants provided written informed consent. A waiver of parental consent was obtained for minors aged 15–17 years old on the conditions that research is minimal risk and parental consent is not a reasonable requirement within the provision of FP services. A NACOSTI licence to conduct research in Kenya was obtained prior to data collection.

All interviewers were female and were trained in safe and sensitive research on violence against women, as per the 2016 WHO guide “*Ethical and safety recommendations for intervention research on violence against women.*”^25^ They were overseen by a senior researcher with over two decades of experience in qualitative research on violence against women and children. Protocols to ensure safety of participants included primarily protecting clients' confidentiality by conducting interviews in a location of visual and auditory privacy, contacting participants to invite them to interviews using general and discreet language, and removing all identifying details from transcripts prior to analysis. Additionally, participants were offered referral resources for GBV support services.

### Analysis

Our analysis applied both deductive and inductive methods using multi-stage, thematic content analysis.^26^ Prior to transcript analysis, codebooks were drafted with structural themes for clients and providers based on the study aims, research questions and hypothesised themes informed by the prior US-based ARCHES qualitative evaluation study.^27^

Once translated transcripts were received and reviewed, they were uploaded to Atlas.ti (version 22.0.2) for coding. Coding was completed by four English-speaking, US-based researchers, including one undergraduate student, two graduate students, and a master’s-level project manager. The process was overseen by the study PI, who has over 30 years of experience in research, including qualitative methods for intervention design and delivery. Each transcript was coded by two analysts who met weekly to resolve coding discrepancies. Differences that were not resolved were assessed in group meetings at which all analysts and the PI participated. In these meetings, summaries of thematic results and observations by individual analysts were also presented. Analyst pairs were rotated to ensure all four analysts were coding consistently. The code book, along with updates and decisions, was also presented in weekly team meetings with all research team partners for input and validation.

Codebooks were modified as analysts began line-by-line coding. Codes were added for emergent themes. Definitions of structural theme codes were clarified, and examples were added. The final code book included 10–12 “parent” codes and approximately 50 “child” codes covering outcomes experienced by providers and clients, reactions to the ARCHES elements, suggested modifications, feasibility, acceptability, deviations from the expected intervention, and barriers to implementation. Transcripts coded with earlier versions of the codebook were re-coded with the final codebook.

Once coding was complete, analytic memos were developed separately for clients and providers according to the major components of the intervention. A final analytic memo was compiled comparing themes from both clients and providers, which served as the basis of the results for this publication. Quotes illustrating key themes were extracted, labelled with the participant's unique ID, which was linked back to their de-identified demographic data and survey responses. Major themes and diverse cases were reported. The final code book and analytic memo were shared with partners and key providers in Nairobi, but not with participating clients, for validation and input. The paper was constructed according to COREQ guidelines. To preserve the anonymity of study participants, quotes are presented labelled with the participant type (client or provider, age, if they reported currently experiencing RC or IPV on their baseline survey or immediate post-intervention survey, and the contraceptive method they received).

### Reflexivity and positionality statement

The lead author is a white, US-based female graduate student with over a decade of experience in sexual and reproductive health and rights and gender-based violence prevention. The authorship team includes individuals at various career stages, ranging from graduate trainees to mid-career professionals and senior researchers. The team reflects diverse racial, gender, and geographic identities, including white, South Asian, and Latino US-based colleagues, and two Kenyan nationals. We acknowledge the influence of our positionalities, particularly given that much of the analysis and authorship team is comprised of Global North researchers, and engaged in reflexive discussions throughout to mitigate bias and elevate local perspectives.

## Results

Clients participating in qualitative interviews were, on average, 27 years old (range 18–40) and had two living children (range 0–6). All participating clients received a contraceptive method, the most common being pills, followed by injections and implants. Every participating client reported prior RC in the past three months on their baseline survey, and 40% reported IPV in the past three months (75% in their lifetime). Of these women reporting recent RC/IPV, 72% disclosed RC to their provider, and 86% disclosed IPV. Regarding implementation, 83% of clients received both RC and IPV screening, 100% received information on covert contraceptive use, and 92% were offered the educational mini-booklet. Of those who disclosed IPV to the provider, 33% were offered a warm referral over the phone to IPV counselling services, though none accepted (Table 1). Of the 12 providers interviewed, 8 providers were female, and 4 providers were male. No other sociodemographic data from providers were collected.

**Table 1.** Client participant characteristics & ARCHES intervention experience

Characteristics	*n* (%)
Age^†^	27 (5.42, 18–40)
Number of children^†^	2 (1.44, 0–6)
FP method received	
Pills	18 (51%)
Injection	9 (26%)
Implant	7 (20%)
Emergency contraception	1 (3%)
RC positive (past 3 months)	35 (100%)
IPV positive (past 3 months)	14 (40%)
ARCHES intervention elements received	
Received RC screening	29 (83%)
Disclosed RC to the provider*	25 (86%)
Received IPV screening	29 (83%)
Disclosed IPV to the provider*	12 (86%)
Counselled on discreet FP methods and/or coping strategies	35 (100%)
Received referral for IPV**	4 (33%)
Offered booklet	33 (92%)
Offered to engage male partner	20 (57%)

^†^ Mean (standard deviation, range).

*Proportion of those who were positive on the baseline survey and screened by the provider.

**Proportion of those who disclosed IPV.

### Structural and emergent themes

Structural and emergent themes were identified for clients and providers. Client structural themes included hypothesised client outcomes (e.g. improved FP use, IPV support awareness) and reactions to receiving ARCHES. Emergent client themes included unanticipated outcomes and concerns related to deviations from ARCHES protocols. Provider structural themes included hypothesised outcomes, perspectives on ARCHES feasibility and acceptability, and suggested modifications, while emergent themes included unanticipated outcomes. Results are presented by intervention components and conclude with quality of care. Client and provider themes are presented together, and disagreements between participant types are noted.

### Information on methods that can be used privately and how to do so

Clients receiving ARCHES found information on covert contraceptive methods from providers to be highly relevant and empowering. Women emphasised that knowing how to use methods privately enhanced their ability to maintain control over pregnancies, even in the face of partner opposition or pressure. Many appreciated that this information supported their contraceptive choices.

“*I felt good when the provider told me how to hide my method because when you go to most of these other hospitals, they are just quick to insert the method but offer no encouragement [for how to use a method in the face of RC] … after inserting the implant on me, he [the ARCHES provider] told me how to keep it hidden. Now, no one at home knows that I am using it. I liked that information.”* (ARCHES client, 19 years old, experiencing RC, received implant)

Several clients also noted that this information could reduce household conflict over contraceptive use.

“*I liked the information because I learned that I may have wished to use the implant and yet your husband does not want you to use it. This causes quarrelling at home. So, I liked it when the provider told me that the three-month injection would be difficult for my husband to discover … Before I had received that counselling, I may get the method and leave the card lying [around the house]. When the husband comes across it, this will cause chaos in the home. So, it is good to receive counselling on how to use the family planning [covertly] and how to hide the cards properly.”* (ARCHES client, 22 years old, experiencing RC, received injection)

Clients valued comprehensive counselling, which dispelled myths (e.g. the implant is too painful) and provided information for them to consider covert methods. Providers’ advice on concealing contraceptive use, such as removing pills from packaging or hiding items, was particularly helpful.

“*I liked that advice that the provider gave me about removing those pills in that packet because I didn’t know that you can remove them. I thought that if you removed them, they could get spoiled.”* (ARCHES client, 29 years old, experiencing RC, received pills)

Overall, women’s stories highlighted that receiving information on how to use contraceptive methods discreetly was essential for them to accept and successfully use a contraceptive method without becoming pregnant.

“*Previously, I could not have used that method [because my partner was pressuring me to have a baby]. It was after the provider talked to me that I opted for that [covert] method. Had he not talked to me and given me the injection, I would most likely be pregnant currently or with another child.”* (ARCHES client, 33 years old, experiencing RC and IPV, received pills)

Providers also felt more confident after ARCHES training, recognising the importance of supporting women’s autonomous contraceptive choices. Most reported positive client responses and noted an increase in the uptake of long-acting methods like IUDs and implants.

*“It has boosted my confidence. I used to think that family planning should be discussed by the couple and they settle for one method of family planning. But after training I realized that its the right of the person who is taking the method. So, the training has affected me positively and has boosted my confidence in offering the services.”* (ARCHES provider, male)

*“[RC] coping strategies and using methods privately have really worked well, because most women did not know that they can still continue using what they prefer. [By] telling them that [about] the coping strategies; we have seen an increased uptake of long-term methods, like IUD and implants.”* (ARCHES provider, female)

However, some clients and providers expressed doubts about the feasibility of certain private-use methods. Implants, for example, were often detectable because they are visible in the arm, leading to concerns about their effectiveness as a covert option. Some clients were also concerned about IUDs and partners detecting the strings. Client concerns over IUDs and implants were substantially more prominent in women’s transcripts over age 25, possibly due to difficulties in hiding these methods when living in close quarters and/or engaging in regular sex compared to casual dating.

*“The only thing we could change was … come up with a different strategy for offering the Implanon [implants]. Most husbands are aware of it [implants].”* (ARCHES provider, male)

“*For that one of the hand [implant] …  even if you wear long sleeves during the day, when you are sleeping at night, he will still be able to know because that scar can be seen.” (*ARCHES client, 36 years old, experiencing RC, received pills)

Despite these concerns, most participants found the ARCHES counselling and strategies valuable for supporting their reproductive autonomy.

### Asking about experiences of RC and IPV

Clients reported that the screening for RC and IPV was helpful and welcome. Many women felt being asked about these issues helped them recognise their abuse experiences as health problems. Providers reported that even clients without prior RC or IPV experiences valued the screening and information for themselves and other women they knew. Clients highlighted that the supportive and non-judgemental provider response fostered trust and a sense of care.

*“In many families, there are women being harassed by their partners. So, when they come and are asked about it [IPV], just like I received information on where I can go for help and report on the abuse and harassment, they will be helped. You get information on what to do, what step to take, you receive help. If they are not asked this, then they cannot be helped and so they remain in fear, intimidated. They can’t speak out.”* (ARCHES client, 22 years old, experiencing RC, received pills)

*“I liked being asked by the provider whether my boyfriend ever hurt me because I felt like she cared about me … * *That she cared about the kind of partner I have even before offering me the family planning.”* (ARCHES client, 18 years old, experiencing RC, received pills)

Providers shared that screening for RC and IPV increased their confidence to address these issues, improved their ability to identify affected clients, and raised awareness of RC and IPV prevalence. They also explained how the ARCHES program helped women recognise abusive situations and provided tools to support them.

*“Through counselling, we realized that so many [women] were experiencing reproductive coercion though they don’t know it. So, when we give them the information through counselling and ask them those questions, they then understand that they have been facing that problem for a long time.”* (ARCHES provider, male)

Some providers noted that time constraints were a challenge, especially when clients disclosed IPV in busy clinic settings, as providing a supportive response required additional time. However, despite these challenges, providers ultimately found the screening process valuable, necessary, and feasible, with both clients and providers recognising its importance in FP care.

### GBV services information and referral

Regarding referral for GBV services, we found mixed opinions. Many clients appreciated the offer of information about IPV support services and felt that knowing about options for support and reporting of abusive partners was empowering. Both clients and providers, however, explained that women were hesitant to accept referrals because of fear of lack of confidentiality, backlash, and/or cultural norms. In younger women’s interviews, concerns and fear around seeking GBV support services were particularly pronounced.

*“You see … they think that even when they are beaten it is okay. … .since the man is paying your rent, is feeding your children and is also paying school fees for your children so they feel that is okay. So for the referrals, very many of them … they don’t go.”* (ARCHES provider, female)

*“For me, that aspect didn’t work quite well. We give assurance that the confidentiality will be maintained but the clients want that confidentiality to be maintained by one person. They don’t want it to go to another person. And as they spoke to that person on the phone, some were not sure what will happen. Is the husband going to be arrested? There was fear of the unknown.”* (ARCHES provider, male)

*“You know if I was to go to Makadara [GBV service] right now; I wouldn’t even know where to start from … I know where it is, yes. I even gave birth to my baby from there. But if I was to go for treatment, I wouldn’t even know where to start from. I just know where the maternity is.”* (ARCHES client, 24 years old, experiencing IPV and RC, received implant)

Some providers mentioned that having the option of these referrals allowed them to feel more confident asking women about their IPV experiences and that the warm-referral mechanism was an improvement over the prior paper-based referral system.

*“This has given me the confidence to handle IPV clients. Initially, I wouldn’t know what to do with a person who was experiencing violence. I didn’t know about counselling them and referral. Now this is referring them to a specific place and you actually give them a phone to talk to the person … When they also get to talk to the other person on the other end, this makes the client to trust you more.”* (ARCHES provider, female)

### Offer of palm-sized, educational mini booklet and information sharing

All clients, regardless of RC/IPV disclosure, were offered the palm-sized, mini-booklet. Clients highlighted the value of the booklet, which contained information on RC, IPV, FP methods, and referral resources. One client noted that it raised her awareness of her rights to a safe relationship and available support services. Many emphasised its importance for retaining information after leaving the clinic, as they might forget or misunderstand what the provider shared without it.

“*I just liked the booklet because through it he had given me information. So I could go and analyze it in detail and get the full information on family planning. You see, what you are told orally you can easily forget, but with the booklet, you can read it on your own … * *Whenever you read it you will add to the knowledge you have. The booklet was important to me because I realized that I have a right to family planning.”* (ARCHES client, 19 years old, experiencing RC, received implant)

Many clients used the booklet to share information with friends, family, and, surprisingly, male partners. One client even suggested that the provider give “*as many [booklets] as possible*” to read and “*share with all those that need it.*” Sharing the booklet helped women access care at the facility, use contraceptive methods discreetly, and in some cases, changed male partners’ perspectives on family planning. One woman even used information on IPV police support to confront her abusive partner, leading to a positive change in his behaviour.

“*I went and faced him … and told him, ‘You beat me up last time, hit my ear and damaged my eardrum, now you have injured my eye. If you beat me another day, I will report you somewhere. I will see you are jailed or ensure you are punished in a way that you will never do this to me or anyone else.’ Since then, he has not repeated it.”* (ARCHES client, 26 years old, experiencing RC and IPV, received injection)

Providers echoed the booklet’s value, noting that it helped spread critical information to women unable to visit the clinic. Clients eagerly shared the booklets, becoming informal advocates in their communities. Providers also observed an increase in clinic referrals from women who received the booklet from others.

*“In fact, when we talked of this [mini-booklet], this lady was very happy and she even intended to circulate all the information to others.”* (ARCHES provider, male)

*“When you issue them [women] with the booklet, they too become advocates or ambassadors for gender based violence … They will tell you that ‘yes I know [of someone] and will take this booklet to them so that they can access this service.’”* (ARCHES provider, male)

### Male partner engagement: securing confidentiality to deliver ARCHES & offering to engage the male partner in FP counselling

The ARCHES approach trained providers to ensure confidentiality for female clients, including separating them from accompanying male partners or family members for private delivery of counselling. This created a safe space to discuss discreet contraceptive use, RC and IPV. At the end of the session, women were told by providers that they could invite their partners or family members for further FP education if they wished.

Providers reflected that ARCHES training improved their practices, emphasising confidentiality and strategies to excuse male partners for private consultations with female clients, counter to culturally normative practices to include male partners in FP consultations. They also learned to schedule follow-up appointments to allow for private counselling when it was difficult to excuse partners.

“*In terms of the counselling I can say that before ARCHES training came …  confidentiality may not have been well observed. But when we went for ARCHES training, it was advised that it was the first thing that you do to a client; to assure the client of complete confidentiality … So now, I am able to provide confidentiality in the best way.”* (ARCHES provider, female)

*“With the training of ARCHES, now we know how to get rid of [excuse] the male partner … Before ARCHES, we would think that they are together, they are a loving couple. But then we came to realize after the ARCHES training, not everyone accompanied by a male partner is supported. … You can say that you want to examine her and take her to another consultation room, then there she will feel free to tell you her experiences or what she is going through.”* (ARCHES provider, female)

Additionally, some providers reflected how the training helped them manage cases where a partner came to demand the removal of a woman’s method, without compromising her agency.

*“I am more confident. … with ARCHES, I have known that I don’t have to give all the information when the male client is in the room. But I can give another appointment … When I meet a client with IPV issues or RC issues, I know how to handle and how to refer. But initially, I wouldn’t know where to refer them to or we would just remove the FP if he [the husband] wanted to. But now with the training, you tell them [women] that you can keep it [the FP method], if that is your choice, so that you can continue using it.”* (ARCHES provider, female)

Some providers noted challenges when male partners resisted leaving the room, limiting their ability to provide confidential ARCHES information. Providers were trained not to administer ARCHES in such situations for the safety of the client, and providers reported utilising this protocol.

*“There was this couple who were a bit older. He said, ‘This is my wife. Why should I leave? Anything that you want to do in private, there is no privacy. This is my wife.’ So, he said no, and that we should just examine her when she is there. So, for this lady, we just gave the general family planning and gave her another later date of return. So, we just issued a one-month injection and told her to come back later, unaccompanied.”* (ARCHES provider, female)

Overall, providers reported strengthening their implementation of counselling alone and client-centred care as a result of the ARCHES training.

Clients largely appreciated the emphasis on privacy and confidentiality, and had mixed feelings about involving male partners. Some felt partner engagement could help dispel FP myths, such as one client whose partner believed FP methods caused diseases like cancer.

“*I liked that it was acceptable for him to accompany me but as things are, it’s difficult and [me] explaining it to him will cause problem. He simply believes that the methods cause diseases like cancer and so says that he cannot allow it since it will cause me problems in future.”* (ARCHES client, 26 years old, experiencing RC and IPV, received injection)

However, many women opposed involving partners, citing concerns about secrecy and fear of adverse reactions at home. Women with recent RC and related IPV experiences and were particularly wary, noting that partner involvement could provoke further violence.

“*I didn’t quite like being told that I could come with him … you know there are things that we usually want to do without anyone knowing, in secret without him knowing. There are those [men] who can never agree. If you bring him here, later it will cause problems at home. He will pretend while here but when you go back home, there will be issues.”* (ARCHES client, 26 years old, experiencing RC and IPV, received injection)

Themes of hesitation around involving male partners were more commonly expressed by women over the age of 25 in our sample than by younger women. Notably, several of these adult women – particularly those seen by male providers – described feeling pressured to involve their partners, which appeared to shape or intensify their hesitations. In some cases, male providers framed partner engagement as essential, leading women to feel pressure to include their partners even when it conflicted with their preferences. These experiences run counter to ARCHES protocols, which emphasise private counselling and involving male partners only when the female client indicates it would be helpful and safe. That this theme was more common among adult women may reflect provider assumptions that adult clients are more likely to be in partnered relationships. While we did not conduct a facility-level analysis, we note that one clinic had a higher number of male providers, which may have contributed to a greater occurrence of these reports from clients at that site.

### Overall quality of care and client–provider relationship

Clients and providers highlighted how ARCHES strengthened their interactions and built trust. Providers explained that trust begins with assurances of privacy and confidentiality as clients enter the clinic and deepens as providers acknowledge women’s challenges with RC and IPV while supporting their FP choices. Some providers noted that even hesitant clients would often return later to share their experiences more openly.

*“Once you give them ARCHES, some of them [female clients] come to realize that they wanted to share because you have shown [you are] caring. When they come back they tell you, ‘Doctor the last time you talked to me there was something you asked me. This is what I have been going through.’ It has enabled us to have a good rapport and to get more information from the client.”* (ARCHES provider, male)

Clients frequently described providers as caring and kind, which made them feel safe discussing their FP desires and challenges. Many clients expressed confidence in returning to the clinic for future help with RC or IPV issues.

*“The provider was being open. She was talking to me as if she was my mum. She was telling me that if I am not happy about anything that she is telling me, I should tell her and she will teach me about another method which I can use to hide. It is not like those providers who will tell you, ‘if you don’t want that one, then leave it alone, I will give you another one because you are wasting my time.’ It is like she was saying, ‘I am here for you.’”* (ARCHES client, 23 years old, experiencing RC, received pills)

“*At least here, the nurse talks to you nicely. Like now, if something like that happens to me, I can call those numbers she gave me or I can come back here and tell her that; ‘I lost that book, give me another one because that problem has happened to me.’ And she will help you.”* (ARCHES client, 22 years old, experiencing RC and IPV, received injection)

Many clients, however, also expressed a desire for more time and detailed information from providers about contraceptive methods, indicating that the allotted time often felt insufficient to fully address their needs.

## Discussion

This study demonstrated that the ARCHES model, as adapted for private clinics in Nairobi, was highly feasible and acceptable to both clients and providers. Clients valued its integration into FP care, emphasising its empowering effects on reproductive health, awareness of violence, and their rights to control contraceptive decisions and seek support for abuse. Providers reported increased confidence and ability to address RC and IPV within FP services, which enhanced the overall quality of care. These findings mirror previous results from US-based evaluations, such as in reproductive health clinics in Pennsylvania.^27^

Notably, we found that information on covert contraceptive use was useful to women. Clients and providers reported that this knowledge often felt novel and enabled women to regain agency over their contraceptive choices, even in constrained environments where covert use was necessary. Prior research supports covert contraceptive use as a “middle ground” of reproductive agency, enabling women to exercise control over fertility in the absence of partner support.^5,7,28^ This aligns with our findings, which suggest that ARCHES effectively bridged gaps in women’s ability to access FP methods independently and was highly acceptable to clients. Other studies have noted that women expect providers to support them in their decisions to use contraceptive discreetly,^29^ which we also found. We also identified instances where women felt that counselling on how to use contraceptives covertly successfully reduced conflict in their home, offering a possible mechanism for how ARCHES may reduce IPV that should be explored in future empirical research.

Screening for RC and IPV, coupled with a supportive, rights-based response, increased women’s critical awareness of abuse and its health impacts. While not all women who experienced IPV sought IPV support services, the knowledge of their availability was empowering, with some women confronting abusive partners and referencing potential consequences for abusive behaviour. These outcomes align with findings from recent work by programmes such as HERA and Safe to Tell, which highlight the importance of framing IPV interventions within the broader context of RC and women’s reproductive agency.^30,31^ Linking RC to IPV also increased feasibility and acceptability for providers, who saw it as addressing clients’ felt contraceptive needs – a known challenge in routine-care GBV screening interventions.^32^ Time was a stated limitation for providers in responding to IPV-positive clients. Thus, in future adaptations, task shifting of in-depth counselling and survivor support to onsite or telehealth social workers should be explored.

Despite the value of GBV referral information, barriers to seeking external IPV services persisted. These included fears of partner arrest for GBV without consent and discomfort sharing personal experiences with new individuals. Similar challenges have been documented in prior studies,^33^ underscoring the need for innovative strategies such as clinic-based social workers or phone-based counselling support to lower these barriers.

Male partner engagement proved challenging for both clients and providers, with women expressing mixed opinions on its appropriateness. Providers occasionally faced difficulties balancing ARCHES protocols with cultural norms or partner resistance. This underscores the importance of maintaining client privacy and autonomy in FP counselling, ensuring women are consulted directly about involving partners and are never pressured to do so. Global FP guidelines should reinforce this approach to protect women’s confidentiality and autonomy. Future adaptations of ARCHES should also consider strengthening this aspect of the provider training and mentorship support.

The findings should be interpreted with several limitations in mind. Results from the three private clinics in Nairobi may not transfer to other populations in Nairobi or Kenya. Selection bias is a possibility, as the study included only a subset of clients and providers from intervention clinics, with purposive sampling favouring clients who experienced key intervention elements. Recall bias is another limitation, given the three-month post-intervention period. Additionally, the absence of data from control clinics prevents direct comparison with clients and providers receiving standard care. Finally, the main analyst team was primarily US-based. While Kenyan partners validated all results, it is possible that greater involvement of Kenyan researchers in the coding and interpretation would have improved or changed results. These factors suggest the need for cautious interpretation of results.

## Conclusion

This study highlights the high acceptability and potential of the ARCHES model to enhance FP care by addressing RC and IPV within private clinics in and around Nairobi. By empowering women with information on covert contraceptive use, raising awareness of abuse, and improving provider capacity to address sensitive issues, ARCHES demonstrated a unique ability to integrate GBV and FP services effectively. To maximise impact, future iterations should explore strategies to overcome barriers to service uptake, expand client access, and ensure sustained adherence to client-centred counselling principles. Findings contribute to a growing body of evidence supporting the integration of FP and GBV interventions to improve women’s reproductive agency and health globally.

## Provenance statement

This article was not commissioned and went through external peer review.
